# Prediction of the occurrence of calcium oxalate kidney stones based on clinical and gut microbiota characteristics

**DOI:** 10.1007/s00345-021-03801-7

**Published:** 2021-08-24

**Authors:** Liyuan Xiang, Xi Jin, Yu Liu, Yucheng Ma, Zhongyu Jian, Zhitao Wei, Hong Li, Yi Li, Kunjie Wang

**Affiliations:** 1 Department of Urology, Institute of Urology (Laboratory of Reconstructive Urology), West China Hospital, No. 37, Guoxue Alley, Chengdu, Sichuan Province China; 2 Department of Biostatistics, University of Michigan, 1415 Washington Heights, Ann Arbor, MI USA; 3 Department of Clinical Research Management, West China Hospital, No. 37, Guoxue Alley, Chengdu, Sichuan Province China

**Keywords:** Kidney calcium oxalate stones, Gut microbiota, Clinical data, Prediction model, Random forest

## Abstract

**Purpose:**

To predict the occurrence of calcium oxalate kidney stones based on clinical and gut microbiota characteristics.

**Methods:**

Gut microbiota and clinical data from 180 subjects (120 for training set and 60 for validation) attending the West China Hospital (WCH) were collected between June 2018 and January 2021. Based on the gut microbiota and clinical data from 120 subjects (66 non-kidney stone individuals and 54 kidney stone patients), we evaluated eight machine learning methods to predict the occurrence of calcium oxalate kidney stones.

**Results:**

With fivefold cross-validation, the random forest method produced the best area under the curve (AUC) of 0.94. We further applied random forest to an independent validation dataset with 60 samples (34 non-kidney stone individuals and 26 kidney stone patients), which yielded an AUC of 0.88.

**Conclusion:**

Our results demonstrated that clinical data combined with gut microbiota characteristics may help predict the occurrence of kidney stones.

## Introduction

Nephrolithiasis is a common urological disease, with a constantly increasing prevalence in recent years. Calcium oxalate stones, which accounts for about 80% of kidney stone types, is the most common category of kidney stones [1]. The calcium oxalate stone pathogenesis often includes a high concentration of oxalate ions, which, by combining with calcium ions or other cations in the urine produces small crystals that adhere to the renal tubular epithelial cells of the kidney, cause a series of reactions, such as inflammation and oxidative stress. These crystals crystallize, nucleate and grow into kidney stones. Among them, oxalic acid increases urinary calcium oxalate saturation about ten times more than calcium, and a mild increase in urinary oxalate can significantly increase the risk of nephrolithiasis [2]. Urinary citrate binds to calcium and inhibits crystallization, thus reducing stone formation. Urine composition can be used to assess stone risk and monitor treatment response in patients with kidney stones [3].

The gut microbiota is crucial in maintaining environmental homeostasis in the gut. 16S ribosomal RNA(rRNA) sequencing offers more possibilities to reveal the diversity of microbes, as several studies have shown significant differences in the gut microbiota between patients with and without kidney stones [4, 5]. Short-chain fatty acids (SFCA) are health-friendly metabolites, produced by the gut microbiota [6] and through different metabolic pathways [7, 8], that provide an ideal environment for the production of acetate, propionate and butyrate [9]. Huang [10] found that short-chain fatty acids have an inhibitory effect on the oxidative stress and inflammatory response of glomerular lineage membrane cells, and that oxidative stress and inflammatory response is involved in stone formation.

Machine learning has been used to analyze microbiome data to identify disease-related biomarkers. Some well-known machine learning algorithms include k-nearest neighbors, random forest, support vector machines, and linear discriminant analysis, and have found applications in genomics, proteomics, systems biology and many other fields [11]. Some studies have developed predictive models for kidney stone recurrence, but with moderate predictive accuracy [12, 13]. With a total of 806 Chinese patients, Wu [14] identified 300 biomarkers from the microbiome and built a predictive model with a moderate predictive accuracy. Overall, there is a lack of works on prediction of calcium oxalate kidney stones, especially based on Chinese patients. Moreover, it is unclear what methods would be most suitable for the prediction of kidney stones, given a variety of available machine learning methods.

To address these questions, we collected microbial data and clinical data from 180 Chinese patients and explored a variety of machine learning methods for predicting the occurrence of calcium oxalate stones. Applications of machine learning methods may help compare their predictiveness using the criterion of area under the curve (AUC) and identify biomarkers that can inform treatment decisions for calcium oxalate stones.

## Materials and methods

### Subject

Our study was in a case–control setting with subjects recruited by the West China Hospital (WCH) from June 2018 to January 2021. Patients were diagnosed with kidney stones by renal ureteral X-ray, urinary ultrasound or abdominal CT examination, while controls were those without renal colic or subclinical retained stone attacks by abdominal ultrasound. All patients received percutaneous nephroscopic lithotripsy or flexible ureteroscopy, with stone composition confirmed by infrared spectroscopy.

The study was approved by the Research Ethics Committee of the WCH, and informed consent was obtained from each participant. The following types of kidney stone patients were excluded: the main component is not calcium oxalate, calcium oxalate is mixed with other components of stones (such as infectious stones or uric acid stones), urinary tract abnormalities, metabolic diseases (including metabolic syndrome), hyperthyroidism, hyperparathyroidism, and long-term use of drugs that may cause kidney stones. Participants were also excluded if they used antibiotics or immunosuppressants three months prior to stool sampling, or had inflammatory bowel disease, irritable bowel syndrome, gastrointestinal tract infections or digestive tumors, bowel surgery, diarrhea and constipation within one month before stool sampling.

A total of 66 non-kidney stone individuals (NS) and 54 patients with kidney stone (KS) were included in this study as training samples, while additional 60 subjects (34 NS and 26 KS) were sampled for validation. Thus, a total of 180 samples were included for this study.

### Data preparation

Microbial DNA extracted from fecal samples was sequenced with 16S rRNA. OTU analysis was performed on 180 samples using Usearch (version 7.0, http://drive5.com/uparse/), and the RDP classifier algorithm was used to annotate taxonomic information. Following the filtering processes as in [15], we excluded samples which were less than 100 reads and OTUs were less than 10 reads, and discarded OTUs which happened < 1% of all the samples. We calculated the relative abundance of each OTU by dividing its value by the total number of reads per sample. Stool SCFA was determined using gas chromatography–mass spectrometry, and urinary oxalate was tested using liquid chromatography–mass spectrometry.

### Feature selection

With the 16 s rRNA data, we collapsed OTUs to the genus level based on a commonly used approach: we first sum their relative abundances respectively, and then drop any OTUs which cannot be annotated at the genus level. The genera selected by both the LDA effect size (LEfSe) (LDA score > 1, *P* < 0.05) [16] and the hierarchical feature engineering (HFE) [17] were used as candidate features. We performed univariate analysis, including Chi-squared test, t-test and Wilcoxon rank sum test, for feature selection.

### Machine learning

On the training set, we used fivefold cross validation to compare the average AUC in order to assess the predictive performance of support vector machines (SVM), random forest (RF), gradient boosted trees (Gboost), lasso, ridge, elastic net (Enet), k-nearest neighbor (KNN) and linear discriminant analysis (LDA). Using the average AUC as the criterion, we found that RF performed the best. We further used the independent validation set to validate the model performance of RF. Analysis was conducted by Python (version 2.7) and R (version 3.6).

## Results

### Taxonomic analysis of microbiota between NS controls and KS patients

**Fig. 1:**
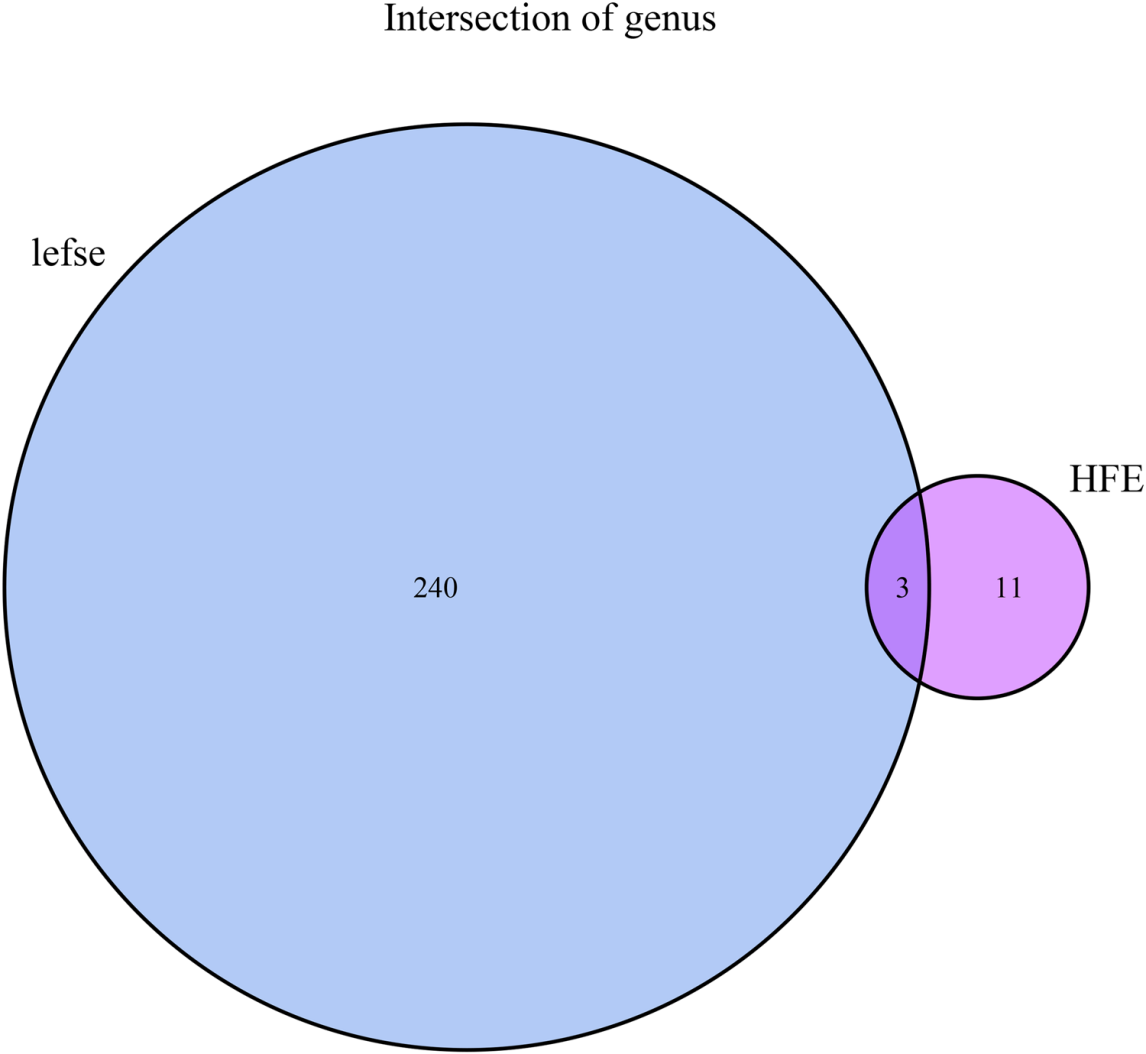
Intersection of genus of LEfSe and HFE

**Fig. 2:**
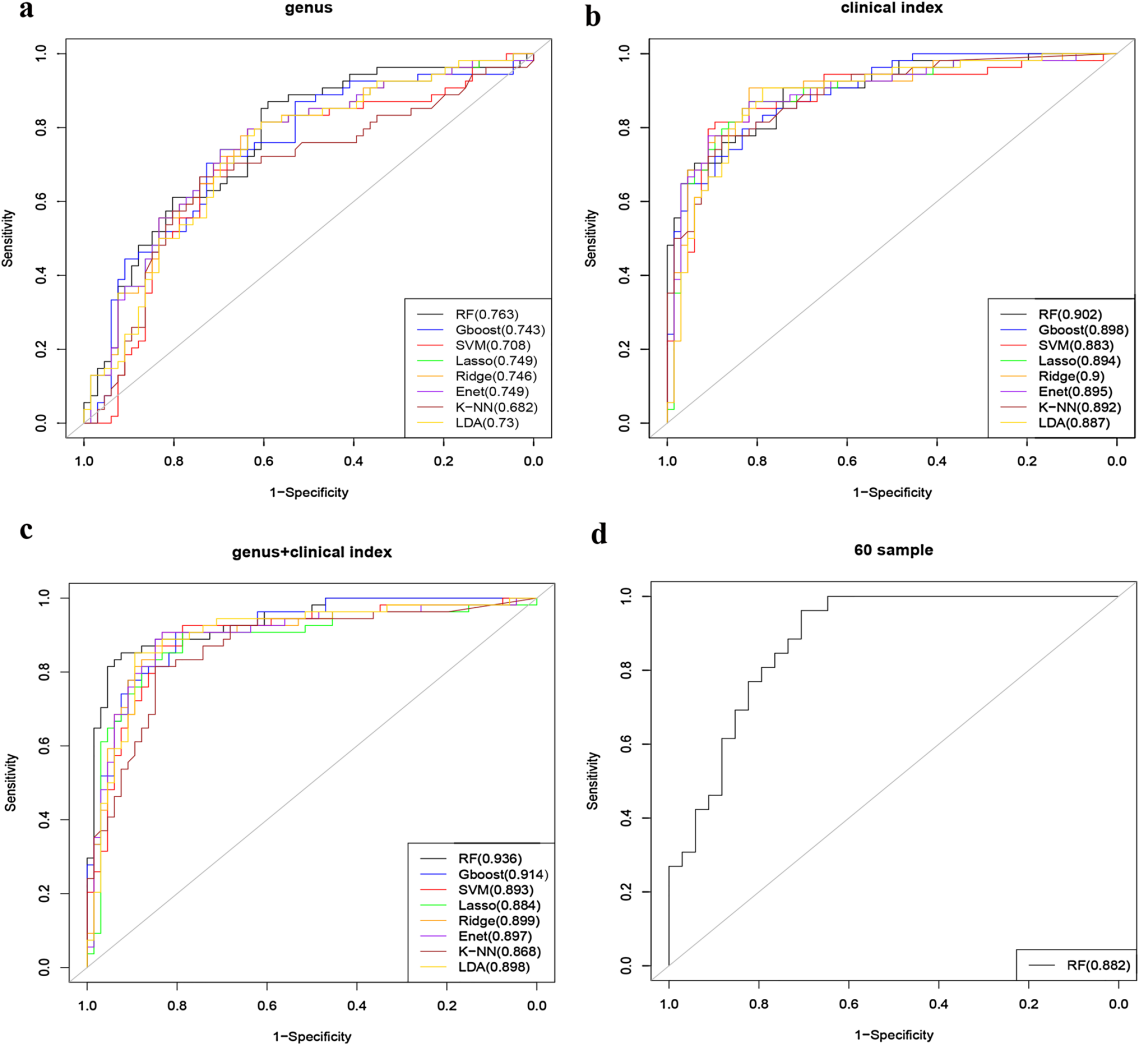
Receiver operating characteristic (ROC) curves were utilized to evaluate the performance of eight methods for predicting kidney stone occurrence (**a** using three genera **b** using five clinical information **c** using three genera plus five clinical indicators). **d** receiver-operating characteristic (ROC) curves were utilized to evaluate the performance of RF for predicting kidney stone occurrence using three genera plus five clinical indicators

The 16S rRNA sequencing data were processed to obtain 5868 OTUs. LEfSe and HFE analysis yielded 243 genera and 14 genera, respectively. The three common genera were: *g__Flavobacterium*, *g__Rhodobacter*, *g__Gordonia* (Fig. 1). Predictive models were built using only these three genus, with AUCs ranging from 0.682 to 0.763 across the eight models (Fig. 2a).

### Clinical characteristics of NS controls and KS patients

In our descriptive analyses, we presented means and standard deviations for continuous variables which were approximately normally distributed; otherwise, we used medians and quartiles. Univariate association analyses revealed no significant differences in age, sex, BMI, propionic acid concentration, isobutyric acid concentration, isovaleric acid concentration, valeric acid concentration, hexanoic acid concentration, calcium concentration and uric acid concentration between NS and KS (Table 1). However, there were significant differences in oxalate concentration, acetic acid concentration, citrate concentration, phosphorus concentration and urinary PH between NS and KS (all *P* < 0.05).

**Table 1:** Characteristics of individuals in the training test

	NS *N* = 66	KS *N* = 54	*p* value
Age	51(48,56)	51(45.5,61)	0.614*
Gender, female (%)	26(39.4)	21(38.9)	0.955#
Body mass index(BMI)	24.28(2.39)	23.86(3.28)	0.442 +
Oxalate(μg/ml)	10.11(4.89, 15.62)	15.62(9.35, 27.88)	0.001*
Acetic acid(ug/mg)	20.79(15.15, 24.32)	43.93(17.59, 57.38)	0.000*
Propionic acid(ug/mg)	0.079(0.055, 0.107)	0.092(0.057, 0.112)	0.650*
Isobutyric acid(ug/mg)	0.007(0.005, 0.012)	0.007(0.004, 0.010)	0.887*
Butyric acid(ug/mg)	0.087(0.061, 0.112)	0.096(0.062, 0.115)	0.650*
Isovaleric acid(ug/mg)	0.006(0.002, 0.011)	0.006(0.004, 0.010)	0.476*
Valeric acid(ug/mg)	0.007(0.001, 0.015)	0.007(0.002, 0.015)	0.728*
Hexanoic acid(ug/mg)	0.0006(0.0004, 0.0011)	0.0007(0.0004, 0.0011)	0.580*
Phosphorus(mmol/L)	15.74( 8.85, 22.71)	11.24(5.68, 16.30)	0.008*
Calcium(mmol/L)	2.30(1.19, 3.87)	1.95(0.91, 4.12)	0.652*
Uric acid(umol/L)	2384(1950, 2788)	1984(1272.5, 2895)	0.260*
Citrate(mg/L)	523.38(255.82, 711.37)	219.95(134.03, 370.59)	0.000*
PH	6.34(5.95, 6.70)	6.77(6.44, 7.14)	0.000*

*Wilcoxon rank sum test

+ *t*-test

^#^Chi-squared test

The predictive models were built based on five clinical characteristics: oxalate concentration, acetic acid concentration, citrate concentration, phosphorus concentration and urinary PH. The random forest model had the highest AUC value of 0.902, while the other models presented AUCs around 0.89 (Fig. 2b).

### Comparisons of prediction models of Genus plus clinical data

We next combined three genus and four clinical indicators for prediction and found the AUC in general improved for all of the methods. Indeed, the AUCs of Gboost, ridge, Enet, LDA and SVM were all above 0.89, except for lasso(0.884) and KNN(0.879), and the RF had the highest AUC of 0.936 (Fig. 2c).

In summary, we found that using the genera data combined with the clinical data produced a more accurate prediction than using the genera or clinical data alone, and random forest produced the best predictive models (Table 2). We next use the validation dataset to further evaluate random forest, which gave an AUC of 0.88 (Fig. 2d).

**Table 2:** AUCs of eight machine learning models

Method	Genus	Clinical	Genus plus clinical
rf	0.7629	0.9018	0.9360
xgboost	0.7427	0.8976	0.9136
svm	0.7082	0.8830	0.8928
lasso	0.7489	0.8939	0.8844
ridge	0.7461	0.8998	0.8993
enet	0.7492	0.8953	0.8973
knn	0.6824	0.8916	0.8678
lda	0.7304	0.8869	0.8976

## Discussion

Comparing eight machine learning methods, we found that random forest outperformed the other machine learning algorithms. Moreover, genera combined with clinical features improved prediction, which suggested that renal stone disease could be diagnosed with clinical indicators in conjunction with gut microbiota data.

Our study identified three disease-related bacteria, among which *g__Flavobacterium* belongs to *Flavobacteriaceae*. The relative abundance of Flavobacterium was reduced in obese patients compared to healthy controls [18]. The other two bacteria, *g__Rhodobacter and g__Gordonia*, belong to the *Rhodobacterace* and Nocardiaceae, respectively. It was reported that some genera of *Rhodobacterace* and Nocardiaceae *Nocardiaceae* can cause infection in humans [19].

Included in our models were oxalate concentration, acetic acid concentration, citrate concentration, phosphorus concentration and urinary pH. Oxalate and acetic acid concentrations are also important indicators of kidney stone occurrence, and higher oxalate is related with a higher risk of calcium oxalate stone [20]. Reducing dietary intake or body synthesis of oxalate is effective in preventing and treating calcium oxalate stones. Acetate is the most abundant SCFA and is an important cofactor for bacterial growth [21, 22]. Citrate can inhibit the formation of CaOx stones [23]. In addition, the pH of urine has been reported to alter several types of stones, including calcium oxalate, calcium phosphate, and uric acid [3]. A study [24] has suggested that urinary phosphorus may play a role in the formation of kidney stones, but not urinary calcium, which agreed to our results that calcium does not differ between patients with stones and healthy individuals.

Random forest is commonly used as an effective classification method in microbiome prediction models. Statnikov [25] used OTUs to perform different classification tasks on eight datasets and found that random forest and support vector machines are the most effective machine learning techniques for performing accurate classification from these microbiome data. Duvallet [15] used the random forest method to classify the 10 diseases and found that for the CRC (colorectal cancer) dataset, the random forest The AUC reached 0.92. Bacteria associated with CRC include *Fusobacterium*, *Porphyromonas*, *Peptostreptococcus*, *Parvimonas*, and *Enterobacter* genera. Pasolli [26] used the microbiota as features to classify five diseases, including cirrhosis, colorectal cancer, and inflammatory bowel disease (IBD), using a random forest classifier. In the cirrhosis dataset, using *Veillonella* and *Streptococcus* genera as features, random forest had AUC of 0.945. In the CRC dataset, *P. stomatitis*, *Fusobacterium nucleatum* and *Streptococcus salivarius* correlated with CRC, the AUC of random forest was 0.873. In the IBD dataset, the AUC was 0.89.

Using discriminant analysis, Chiang [27] utilized 151 calcium oxalate stones patients and 105 healthy controls of four genetic polymorphisms: vascular endothelial growth factor (VEGF), E-calcine adhesion, urokinase, and cytochrome p450c17, as well as relevant environmental factors (milk, water, outdoor activity and coffee consumption), presented a prediction model of kidney stones. The results showed that when only genetic factors were considered, the classification success rate of DA was 64%; but with all relevant factors considered (genetic and environmental factors), the classification success rate for DA was 74%. In [28], an SVM model for detecting kidney stone types by using 42 features of 936 kidney stone patients, including sex, acid urine status, calcium levels, back pain and urinary tract infection, reached an AUC of 86.9%.

To our knowledge, no research has been done to combine gut microbiota with clinical characteristics to predict the occurrence of kidney stones. Filling this gap, we constructed a prediction model of calcium oxalate kidney stones using microbiota, metabolites of microbiota and urinary parameters. Our machine learning results may provide new and non-invasive potential diagnostic biomarkers for calcium oxalate kidney stones.
